# The Epidemiology of Ground Glass Opacity Lung Adenocarcinoma: A Network-Based Cumulative Meta-Analysis

**DOI:** 10.3389/fonc.2020.01059

**Published:** 2020-07-21

**Authors:** Xiongfei Li, Fan Ren, Shuhang Wang, Zhicheng He, Zuoqing Song, Jun Chen, Song Xu

**Affiliations:** ^1^Department of Lung Cancer Surgery, Tianjin Medical University General Hospital, Tianjin, China; ^2^Tianjin Key Laboratory of Lung Cancer Metastasis and Tumor Microenvironment, Lung Cancer Institute, Tianjin Medical University General Hospital, Tianjin, China; ^3^Department of Clinical Trials Center, National Cancer Center, Cancer Hospital Chinese Academy of Medical Sciences, Beijing, China; ^4^Department of Thoracic Surgery, The First Affiliated Hospital of Nanjing Medical University, Nanjing, China

**Keywords:** ground glass opacity, lung adenocarcinoma, cumulative meta-analysis, epidemiological trends, lung cancer screening criteria

## Abstract

**Introduction:** Due to the introduction of low-dose computed tomography (CT) and screening procedures, the proportion of early-stage lung cancer with ground glass opacity (GGO) manifestation is increasing in clinical practice. However, its epidemiological characteristics is still not fully investigated.

**Methods:** We retrieved all solitary GGO adenocarcinoma lung cancer (ADLC) on the PubMed, Cochrane Library, and Embase databases until January 1, 2019 and extracted the general information to perform the meta-analysis, mainly focusing on age, gender, and smoking status.

**Results:** A total of 8,793 solitary GGO ADLC patients from 53 studies were included in this analysis. The final pooled analysis showed that the female proportion, average diagnosis age, and non-smoking proportion of solitary GGO ADLC was 0.62 (95% CI, 0.60–0.64), 56.97 (95% CI, 54.56–59.37), and 0.72 (95% CI, 0.66–0.77), respectively. The cumulative meta-analysis and meta-trend analysis confirmed that the average age at diagnosis has been decreasing while the non-smoking proportion significantly increased in the past two decades.

**Conclusions:** From our epidemiological analysis, it demonstrates that the clinical characteristics of GGO lung cancer patients may be out of the high-risk factors. Therefore, we propose to reconsider the risk assessment and current lung cancer screening criteria.

## Introduction

Due to the introduction of low-dose computed tomography (CT) and screening procedures, the number of diagnoses of pulmonary ground glass opacity (GGO) lung cancer in clinical practice is increasing (1, 2). The GGO manifestation is generally caused by local airspace filling as a result of inflammation or neoplastic proliferation, and some studies reported that the malignancy rate of GGO was 63%, which has a higher malignant potential than solid nodules (3, 4). The GGO manifestation generally correlates with a lepidic, *in situ*, non-invasive growth pattern of cells along preexisting alveolar structures (4). A previous study has reported that GGO lung cancer may have several unique features, including an insignificant association with smoking history and a low degree of invasive biological characteristics (3). As the importance of GGO lung cancer is increasing, more researches have focused on the diagnosis and treatment of this early stage lung cancer; however, the epidemiology of lung cancer with GGO manifestation has not yet been fully elucidated. In this study, we summarized all of the publications concerning solitary GGO adenocarcinoma lung cancer (ADLC) and investigated the epidemiological data of this unique type of lung cancer by the use of a cumulative meta-analysis. The primary outcome is female proportion, and the secondary outcomes are average diagnosis age and non-smoking proportion. All analyses of our study were specified *a priori* in the protocol, and our study was registered and the protocol made available on the PROSPERO (the registration number CRD42019119240).

## Methods

This study was reported on the basis of the Preferred Reporting Items for Systematic Reviews and Meta-Analyses (PRISMA) statement guidelines (Supplementary Table 1).

Two individual researchers conducted the platform searches on the PubMed, Cochrane Library, and Embase databases. Literature retrieving was carried out through a combined searching of subject terms (“MeSH” on PubMed and “Emtree” on “Embase”) and free terms on the platforms of PubMed and Embase, and through keywords searching on platform of Cochrane Library. Detailed searching criteria used in the three electronic platforms are available in Appendix 1.

All available studies that had been published in English until January 1, 2019 on patients with solitary GGO ADLC were included, and the inclusion and exclusion criteria were listed. The inclusion criteria of study were (1) GGO manifestation and (2) finally pathologically confirmed ADLC. The exclusion criteria were the following: (1) studies with a design of literature review, systematic review, basic research, letter to editors, diagnostic study, and so on; (2) studies that include the following cases and cannot be ruled out—multiple GGO, benign GGO, or pure solid nodules; (3) studies that did not involve basic information of patients; and (4) studies using repeated patients cohorts with any other study. There were no limitations on the participants' nationalities.

The Newcastle–Ottawa quality assessment scale (NOS) and National Institute for Clinical Excellence (NICE) quality assessment scale were performed to assess methodological quality and risk of bias for cohort studies and case series studies, respectively. We extracted the general characteristics of GGO patients (amount, age, gender, and smoking status) to perform the meta-analysis. For the proportions of GGO adenocarcinoma of the female gender and the smoking histories, the single rate was determined, and the single mean value was used for the calculation of the average diagnosis ages of the patients. Meta-analysis was performed on all the data using fixed or random effect through heterogeneity, which was tested by estimating value of *I*^2^ (significance level at *I*^2^ > 50%) or using the Cochrane *Q*-test (significance level at *P* < 0.100). The cumulative meta-analysis was also performed, and the trend test was performed to confirm the trend of cumulative meta-analysis, as sorted by years. The methods of Begg's and Egger's regression asymmetry test were performed to test publication bias, and *P* < 0.050 and *P* < 0.100 were considered to be statistically significant publication bias for Begg's and Egger's, respectively, (5). If the *P*-value indicates the existence of publication bias, the non-parametric trim and fill method would be performed to revise the result of meta-analysis (6). Sensitivity analysis was performed by omitting each individual study to check the stability of the result, and studies causing instability would be removed from the meta-analysis. The whole process of data analyses was performed by the software Stata version 13.0 (Stata Corp LLC, College Station, TX, USA).

## Results

**Figure 1:**
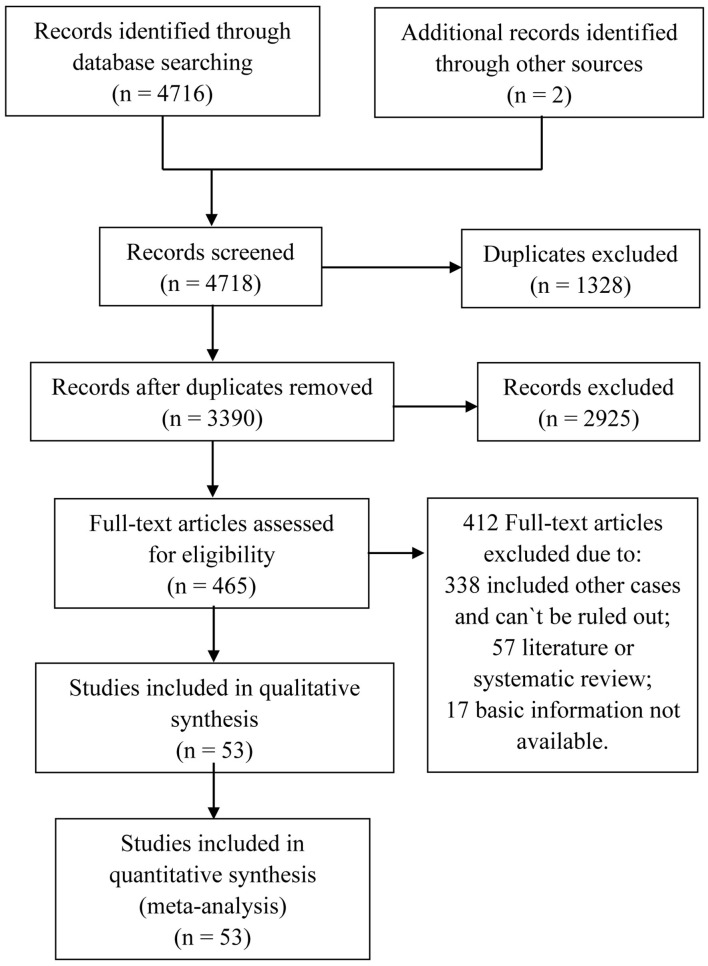
Flowchart of studies identification and selection.

The process of eligible literature selection is presented in Figure 1, and a total of 8,793 solitary GGO ADLC patients from 53 studies until 2019 were recruited in the meta-analysis, mainly focusing on age, gender, and smoking status (7–59). No article was excluded by methodological quality and risk of bias and sensitivity analysis for significant heterogeneity (Supplementary Figures 1–3). The summary of individual study is listed in Table 1. All the meta-analyses were performed with a random-effect model (*I*^2^ > 50%).

**Table 1:** Summary of included studies.

**Author**	**Character of studies**				**Character of patients**					
	**Year**	**Study design**	**Country**	**NOS(star) /NICE**	**Primary tumor**	**Case with GGO**	**GGO rate**	**Female (%)**	**Age(mean)**	**Non-smoking rate**
Kodama et al. (59)	2001	Retrospective cohort	Japan	7	Lung adenocarcinoma	52	0 < *R* ≤ 1	0.52	NA	NA
Matsuguma et al. (58)	2002	Retrospective cohort	Japan	7	Lung adenocarcinoma	57	0 < *R* ≤ 1	0.68	NA	0.65
Suzuki et al. (57)	2002	Case series	Japan	5	Lung adenocarcinoma	69	0 < *R* ≤ 1	0.55	NA	NA
Nakamura et al. (56)	2004	Case series	Japan	6	Lung adenocarcinoma	27	*R* = 1	0.56	66.40	NA
Nakata et al. (55)	2005	Retrospective cohort	Japan	8	Lung adenocarcinoma	101	10 ≤ *R* ≤ 1	0.60	62.76	0.37
Suzuki et al. (54)	2006	Case series	Japan	5	Lung adenocarcinoma	170	0 < *R* ≤ 1	0.60	NA	NA
Park et al. (53)	2009	Case series	Korea	5	Lung adenocarcinoma	44	*R* = 1	0.50	NA	NA
Okada et al. (52)	2011	Retrospective cohort	Japan	8	Lung adenocarcinoma	304	20 ≤ *R* ≤ 1	0.61	65.00	NA
Cho et al. (51)	2013	Case series	Korea	5	Lung adenocarcinoma	28	*R* = 1	0.32	NA	NA
Duann et al. (50)	2013	Retrospective cohort	China	6	Lung adenocarcinoma	46	50 ≤ *R* ≤ 1	0.50	60.28	NA
Lim et al. (49)	2013	Case series	Korea	5	Lung adenocarcinoma	46	*R* = 1	0.43	NA	0.70
Tsutani et al. (48)	2013	Retrospective cohort	Japan	8	Lung adenocarcinoma	299	0 < *R* < 1	0.57	65.70	NA
Uehara et al. (47)	2013	Retrospective cohort	Japan	8	Lung adenocarcinoma	334	25 ≤ *R* ≤ 1	0.61	65.40	NA
Hattori et al. (46)	2014	Case series	Japan	5	Lung adenocarcinoma	112	0 < *R* < 1	0.63	NA	NA
Tsutani et al. (45)	2014	Retrospective cohort	Japan	7	Lung adenocarcinoma	239	50 < *R* ≤ 1	0.61	NA	NA
Zhang et al. (44)	2014	Case series	China	5	Lung adenocarcinoma	43	50 ≤ *R* ≤ 1	0.79	NA	NA
Cho et al. (43)	2015	Retrospective cohort	Korea	8	Lung adenocarcinoma	164	*R* = 1	0.55	61.50	0.65
Cho et al. (42)	2015	Retrospective cohort	Korea	7	Lung adenocarcinoma	71	0.25 < *R* ≤ 1	0.59	NA	NA
Hwang et al. (41)	2015	Retrospective cohort	Korea	8	Lung adenocarcinoma	197	0 < *R* ≤ 1	0.61	61.32	NA
Nakamura (40)	2015	retrospective cohort	Japan	7	Lung adenocarcinoma	25	50 ≤ *R* ≤ 1	0.52	NA	NA
Sakurai et al. (39)	2015	Retrospective cohort	Japan	8	Lung adenocarcinoma	201	0 < *R* ≤ 1	0.57	NA	0.56
Yang et al. (38)	2015	Retrospective cohort	China	6	Lung adenocarcinoma	158	0 < *R* ≤ 1	0.61	56.07	0.76
Choi et al. (37)	2016	Retrospective cohort	Korea	8	Lung adenocarcinoma	288	0.2 < *R* ≤ 1	0.56	59.30	0.68
Hattori et al. (36)	2016	Retrospective cohort	Japan	8	Lung adenocarcinoma	616	0 < *R* < 1	0.62	66.60	NA
Moon et al. (35)	2016	Retrospective cohort	Korea	8	Lung adenocarcinoma	83	*R* = 1	0.63	NA	0.77
Qiu et al. (34)	2016	Case series	China	5	Lung adenocarcinoma	81	0 < *R* ≤ 1	0.68	NA	0.79
Si et al. (33)	2016	Retrospective cohort	China	6	Lung adenocarcinoma	53	*R* = 1	0.85	NA	0.89
Fukui et al. (32)	2017	Retrospective cohort	Japan	7	Lung adenocarcinoma	250	50 ≤ *R* ≤ 1	0.58	63.52	NA
Hattori et al. (31)	2017	Retrospective cohort	Japan	8	Lung adenocarcinoma	177	0 < *R* ≤ 0.5	0.63	66.70	NA
Hattori et al. (30)	2017	Retrospective cohort	Japan	8	Lung adenocarcinoma	262	0 < *R* ≤ 1	0.68	61.03	NA
Moon et al. (29)	2017	Retrospective cohort	Korea	8	Lung adenocarcinoma	52	0.5 < *R* ≤ 1	0.60	NA	0.77
She et al. (28)	2017	Retrospective cohort	China	8	Lung adenocarcinoma	898	*R* = 1	0.65	54.12	0.90
Wang et al. (27)	2017	Retrospective cohort	China	6	Lung adenocarcinoma	67	*R* = 1	0.81	55.81	NA
Zhou et al. (26)	2017	Case series	China	5	Lung adenocarcinoma	137	*R* = 1	0.78	NA	NA
Berry et al. (25)	2018	Retrospective cohort	USA	8	Lung adenocarcinoma	69	0 < *R* ≤ 0.25	0.62	69.00	0.46
Huang et al. (24)	2018	Retrospective cohort	China	8	Lung adenocarcinoma	789	0 < *R* ≤ 1	0.67	61.28	0.77
Kim et al. (23)	2018	Retrospective cohort	Korea	8	Lung adenocarcinoma	202	0 < *R* ≤ 1	0.50	NA	0.73
Kim and Goo (22)	2018	Case series	Korea	5	Lung adenocarcinoma	117	*R* = 1	0.55	NA	NA
Lee et al. (21)	2018	Retrospective cohort	Korea	6	Lung adenocarcinoma	36	*R* = 1	0.69	NA	0.89
Li et al. (20)	2018	Retrospective cohort	China	6	Lung adenocarcinoma	393	0 < *R* ≤ 1	0.70	NA	0.75
Li et al. (19)	2018	Retrospective cohort	China	6	Lung adenocarcinoma	109	0 < *R* ≤ 1	0.68	57.21	NA
Liu et al. (18)	2018	Case series	China	5	Lung adenocarcinoma	48	0 < *R* ≤ 1	0.77	NA	NA
Predina et al. (17)	2018	Case series	USA	5	Lung adenocarcinoma	20	0 < *R* ≤ 1	0.65	NA	NA
Sagawa et al. (16)	2018	Prospective cohort	Japan	7	Lung adenocarcinoma	53	0.8 ≤ *R* ≤ 1	0.53	NA	NA
Su et al. (15)	2018	Retrospective cohort	China	8	Lung adenocarcinoma	245	0 < *R* ≤ 1	0.64	59.33	0.78
Suzuki et al. (14)	2018	Retrospective cohort	Japan	8	Lung adenocarcinoma	160	0 < *R* ≤ 1	0.51	NA	0.44
Wang et al. (13)	2018	Case series	China	5	Lung adenocarcinoma	146	0 < *R* < 1	0.66	NA	0.92
Wang et al. (12)	2018	Retrospective cohort	China	8	Lung adenocarcinoma	165	*R* = 1	0.78	54.20	NA
Wang et al. (11)	2018	Case series	China	6	Lung adenocarcinoma	230	0 < *R* < 1	0.58	NA	NA
Xue et al. (10)	2018	Retrospective cohort	China	6	Lung adenocarcinoma	68	0 < *R* < 0.5	0.69	52.30	0.69
Yagi et al. (9)	2018	Case series	Japan	6	Lung adenocarcinoma	101	0 < *R* ≤ 1	0.55	69.42	NA
Yang et al. (8)	2018	Case series	China	6	Lung adenocarcinoma	51	0 < *R* ≤ 0.5	0.57	69.40	NA
Yao et al. (7)	2018	Retrospective cohort	China	6	Lung adenocarcinoma	40	50% < *R* ≤ 1	0.68	NA	0.78

*Summary of 53 studies with 8,793 patients from the literatures. Newcastle–Ottawa quality assessment scale (NOS) and National Institute for Clinical Excellence quality assessment scale (NICE) were performed to assess methodological quality and risk of bias for cohort studies and case series studies, respectively*.

**Figure 2:**
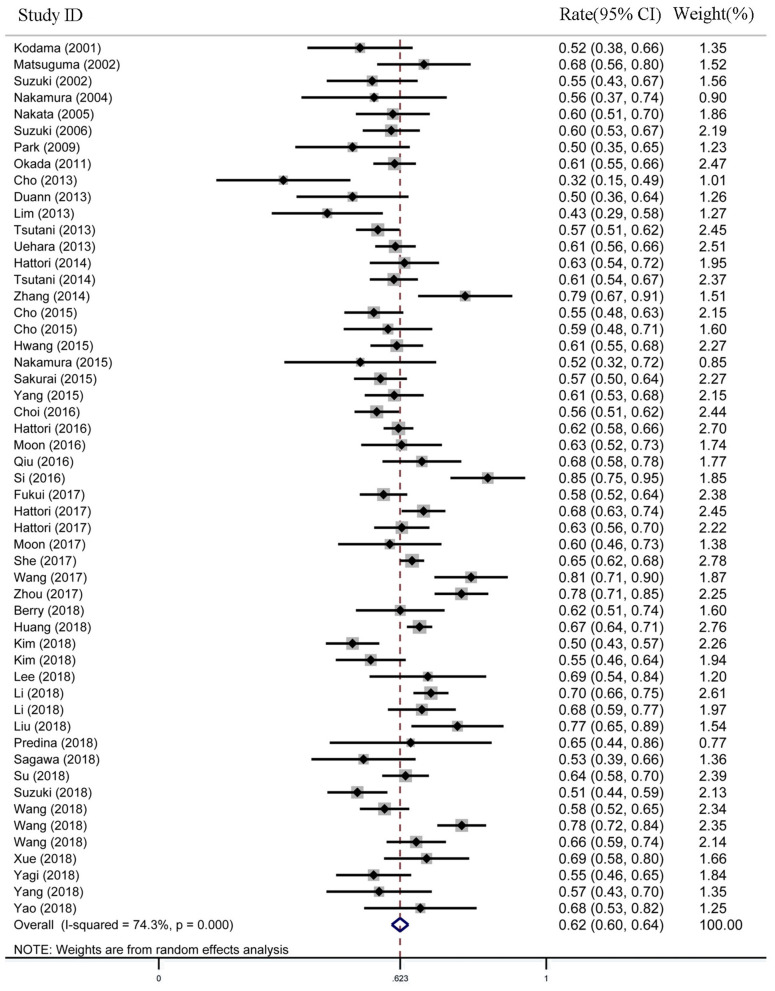
The meta-analysis forest map of the female rate of solitary ground glass opacity (GGO) adenocarcinoma lung cancer (ADLC).

**Figure 3:**
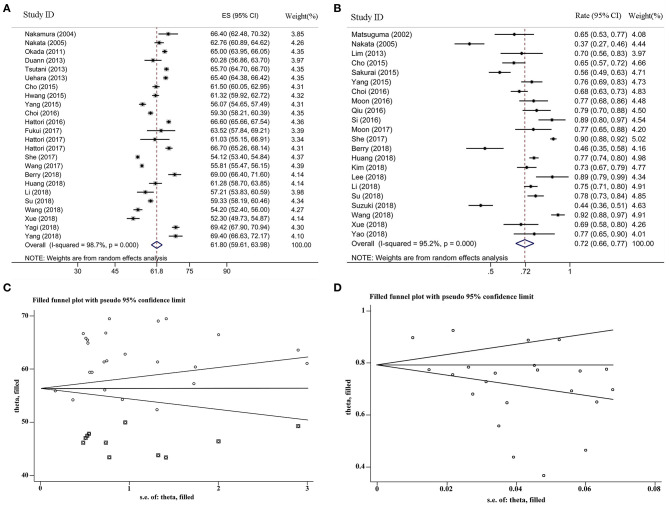
The meta-analysis forest maps, funnel plot with non-parametric trim-and-fill method for solitary ground glass opacity (GGO) adenocarcinoma lung cancer (ADLC). **(A)** The meta-analysis forest map of the average diagnosis age; **(B)** the meta-analysis forest map of the non-smoking rate; **(C)** the funnel plot of average age group with non-parametric trim and fill method; **(D)** the funnel plot of non-smoking rate group with non-parametric trim and fill method.

For the female proportion of GGO ADLC, all 8,793 patients were included in the meta-analysis, and the results demonstrated that the female proportion was 0.62 (95% CI, 0.60–0.64), and the *P*-value of Begg's and Egger's test is > 0.1, indicating that there was no existence of publication bias (Figure 2). For average diagnosis age group, 24 articles involving 5,785 GGO ADLC patients were included for the meta-analysis of age (Figure 3A). The *P*-value of Egger's test was 0.015, which indicated the presence of publication bias, and the non-parametric trim-and-fill method was performed to adjust the effect value (5). Eleven studies were filled to rectify bias, and the final pooled average diagnosis age was 56.97 (95% CI, 54.56–59.37) (Figure 3C). A total of 4,330 GGO ADLC patients from 22 articles were assessed in the meta-analysis for smoking status (Figure 3B). The *P*-value of Egger's test was 0.003, and the non-parametric trim-and-fill method was performed. No studies were estimated to rectify the bias, and the final pooled non-smoking proportion of solitary GGO ADLC was 0.72 (95% CI, 0.66–0.77) (Figure 3D).

**Figure 4:**
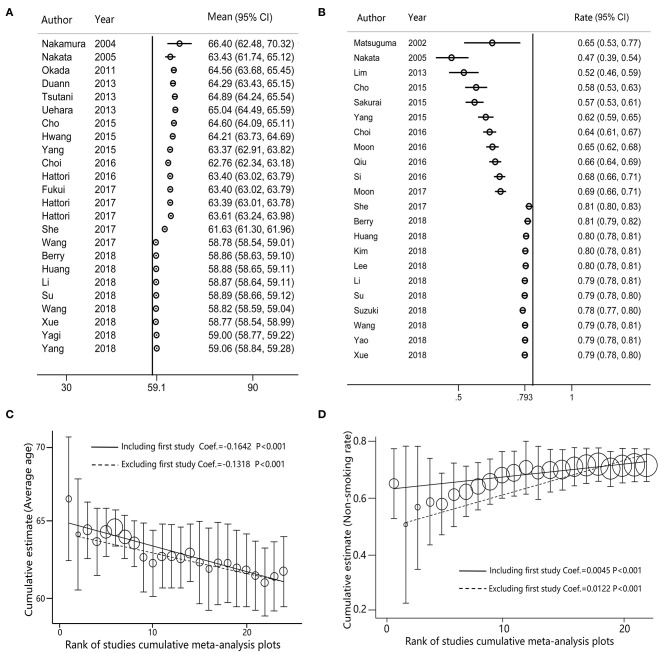
The cumulative meta-analysis forest maps and meta-trend analysis for solitary ground glass opacity (GGO) adenocarcinoma lung cancer (ADLC), as sorted by years. **(A)** The cumulative meta-analysis forest map of the female rate; **(B)** the cumulative meta-analysis forest map of the non-smoking rate; **(C)** the trend analysis for the cumulative meta-analysis of the female rate; **(D)** the trend analysis for the cumulative meta-analysis of the non-smoking rate.

The cumulative meta-analysis of age group demonstrated that the average age had decreased from 66.40 to 59.06 years (95% CI, 58.84–59.28) (Figure 4A), and the meta-trend analysis confirmed that the decrease in age was statistically significant (*P* < 0.001) (Figure 4C). The cumulative meta-analysis of non-smoking group indicated that the non-smoking proportion in GGO patients has increased in the past two decades (Figure 4B), which was statistically significant in the meta-trend analysis (*P* < 0.001) (Figure 4D).

## Discussion

GGO-predominant lung cancers are typically characterized as non-invasively or minimally invasively low-grade adenocarcinomas and had good prognosis after surgical intervention (60). Early detection and therapeutic intervention for these early stage lung cancers is an important opportunity for decreasing overall mortality of lung cancer. Some lung cancer screening criteria have been proposed, which always consider heavy smoking history as a key factor for risk assessment (61, 62). The US Preventive Services Task Force (USPSTF) recommends lung cancer screening among individuals aged 55–80 years with a 30 pack-year cigarette smoking history (61). In addition, the latest Lung Cancer Screening from National Comprehensive Cancer Network (NCCN) Guidelines determines age <50 years and smoking history lower than 20 pack-year as low risk, in which lung cancer screening is not recommended (62). Our meta-analysis indicates that the pooled non-smoking proportion is 0.72. The majority of GGO lung cancer patients are female, and the average age at diagnosis has been significantly decreasing in the past two decades. Our data demonstrate that the clinical characteristics of GGO lung cancer patients may be out of the high-risk factors who are inappropriate for the lung cancer screening. Zhang et al. performed LDCT for 8,329 hospital employees from different regions, and 179 cases were pathologically confirmed lung cancer and 98.9% (171) cases presented with GGO (63). In Zhang's study, there was a higher lung cancer detection rate in female than male patients (2.5 vs. 1.3%), and the lung cancer detection rate of non-smokers was also high than smokers (2.2 vs. 1.4%). In subset analysis by age, the lung cancer detection rates were 1.0, 2.6, and 2.9% in the “age ≤ 40 years,” “40 < age ≤ 55 years,” and “age > 55 years” group, respectively (63). According to this substantial data, Zhang proposed that the “high-risk” population for lung cancer is changing, and more lung cancers from the traditionally “low-risk” groups, such as young female non-smokers, could be detected by LDCT (63). These finding are completely consistent with our study. More and more female younger non-smokers were diagnosed with lung cancer; however, the exact reasons of this phenomenon are still uncertain. Most researchers thought that the phenomenon may be caused by life pressure, living habits, and hormone levels; however, it needs to be further investigated. Luo et al. conducted a cohort study that demonstrated that younger and light smoker patients with lung cancer who are not recommended for screening have similar lung cancer survival to those lung cancer patients who meet all the USPSTF screening criteria (64). This study supports our findings that the individuals with low-risk factors should be concerned as well, and the criteria of current lung cancer screening might not be perfect. However, the cost effectiveness needs to be evaluated if more low risk individuals are included in low-dose computed tomography (CT) screening (65). A limitation of this study is that all of the included studies were retrospective studies that have a lower level of evidence compared to prospective studies.

## Conclusions

Our study demonstrated that the majority of GGO ADLC patients are female with non-or light smoking history, and the average age at diagnosis has been significantly decreasing. This indicates that there are more lung cancers being detected from the traditionally “low-risk” groups, such as young female non-smokers. It is well-accepted that early detection of lung cancers is the most important procedure that contributes to improved survival outcomes and reduced lung cancer mortality. Therefore, we propose that, in order to identify these very early stage GGO lung cancer patients with low-risk factors, it is necessary to reconsider the risk assessment and current lung cancer screening criteria.

## Data Availability Statement

All datasets generated for this study are included in the article/Supplementary Material.

## Author Contributions

XL, FR, and SW retrieved and analyzed all of the data in the study. ZH and ZS revised the manuscript for important intellectual contents. SX and JC designed, checked, and supervised the study process. All authors contributed to the article and approved the submitted version.

## Conflict of Interest

The authors declare that the research was conducted in the absence of any commercial or financial relationships that could be construed as a potential conflict of interest.

## Supplementary Material

Supplementary Figure 1 Sensitivity analysis for female rate of solitary GGO ADLC.

Supplementary Figure 2 Sensitivity analysis for average year of solitary GGO ADLC.

Supplementary Figure 3 Sensitivity analysis for non-smoking rate of solitary GGO ADLC.

Supplementary Table 1 The PRISMA checklist.

Appendix 1 Searching strategies performed for eligible study retrieval.
