# Supplementary figures and images for: The Epidemiology of Ground Glass Opacity Lung Adenocarcinoma: A Network-Based Cumulative Meta-Analysis
Source: Front Oncol. 2020 Jul 21;10:1059. doi: 10.3389/fonc.2020.01059 (PMC7386063; doi:10.3389/fonc.2020.01059)

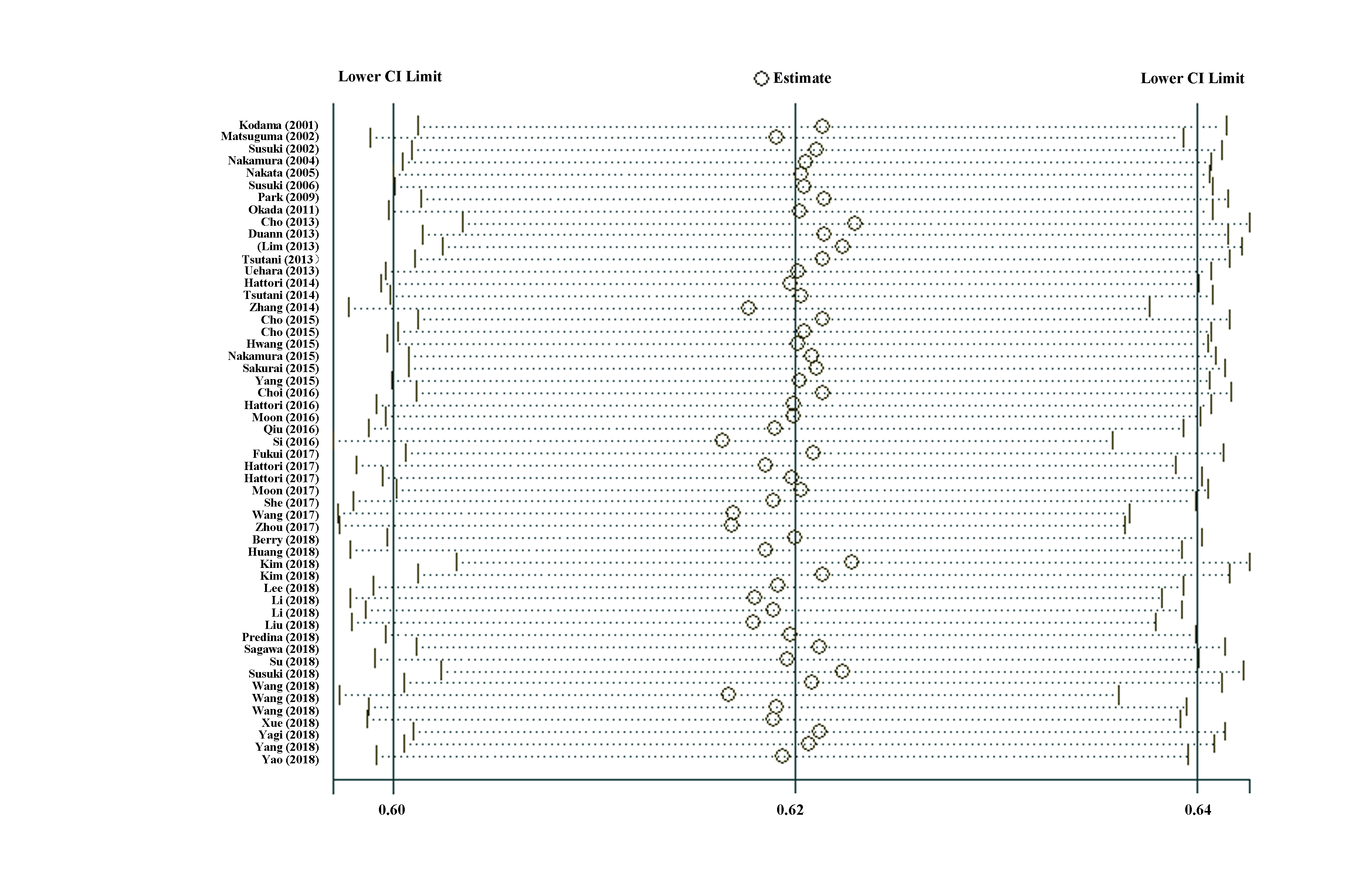

Supplement: Supplementary Figure 1 — Sensitivity analysis for female rate of solitary GGO ADLC. [file Image_1.JPEG]

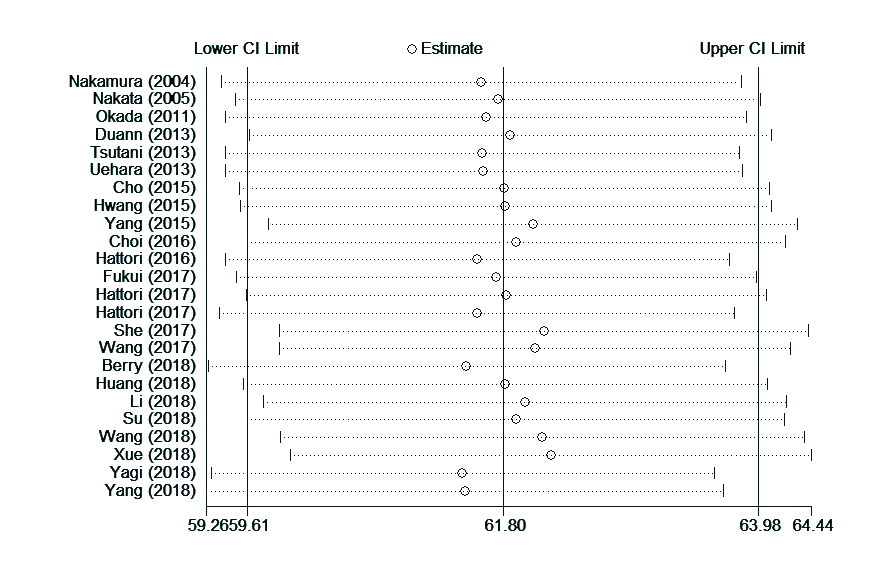

Supplement: Supplementary Figure 2 — Sensitivity analysis for average year of solitary GGO ADLC. [file Image_2.JPEG]

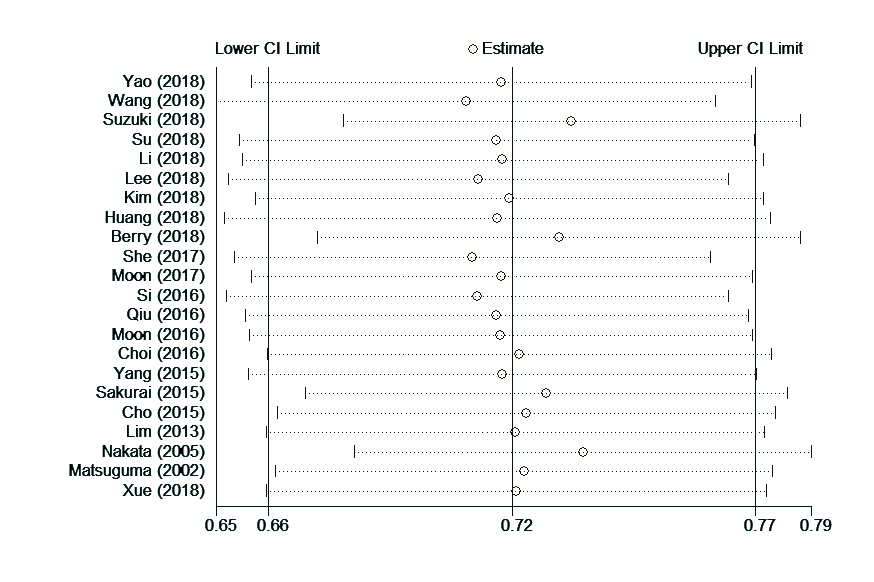

Supplement: Supplementary Figure 3 — Sensitivity analysis for non-smoking rate of solitary GGO ADLC. [file Image_3.JPEG]
