# Supplementary material for: The Epidemiology of Ground Glass Opacity Lung Adenocarcinoma: A Network-Based Cumulative Meta-Analysis
Source: Front Oncol. 2020 Jul 21;10:1059. doi: 10.3389/fonc.2020.01059 (PMC7386063; doi:10.3389/fonc.2020.01059)
Supplement: Appendix 1 — Searching strategies performed for eligible study retrieval. [file Data_Sheet_1.docx]

**Appendix 1.** Searching strategies performed for eligible study retrieval

The searching criteria on PubMed was following:

#1 ((((((((((((((((((Lung Neoplasms) OR Pulmonary Neoplasms) OR Neoplasms, Lung) OR Lung Neoplasm) OR Neoplasm, Lung) OR Neoplasms, Pulmonary) OR Neoplasm, Pulmonary) OR Pulmonary Neoplasm) OR Lung Cancer) OR Cancer, Lung) OR Cancers, Lung) OR Lung Cancers) OR Pulmonary Cancer) OR Cancer, Pulmonary) OR Cancers, Pulmonary) OR Pulmonary Cancers) OR Cancer of the Lung) OR Cancer of Lung)

#2 ((((((ground glass opacity) OR ground glass node) OR ground glass nodule)) OR GGO)) OR GGN

#3 #1 AND #2

The searching criteria on Embase was following:

#1 'lung cancer'/exp

#2 pulmonary AND neoplasms OR (neoplasms, AND lung) OR (lung AND neoplasm) OR (neoplasm, AND lung) OR (neoplasms, AND pulmonary) OR (neoplasm, AND pulmonary) OR (pulmonary AND neoplasm) OR (cancer, AND lung) OR (cancers, AND lung) OR (lung AND cancers) OR (pulmonary AND cancer) OR (cancer, AND pulmonary) OR (cancers, AND pulmonary) OR (pulmonary AND cancers) OR (cancer AND of AND the AND lung) OR (cancer AND of AND lung) OR (lung AND neoplasms)

#3 #1 OR #2

#4 'ground glass opacity'/exp

#5 GGN OR (ground AND glass AND node) OR (ground AND glass AND nodule) OR GGO

#6 #4 OR #5

#7 #3 AND #6

The searching criteria on Cochrane Library was following:

#1 MeSH descriptor: [Lung Neoplasms] explode all trees

#2 (Pulmonary Neoplasms OR Neoplasms, Lung OR Lung Neoplasm OR Neoplasm, Lung OR Neoplasms, Pulmonary OR Neoplasm, Pulmonary OR Pulmonary Neoplasm OR Lung Cancer OR Cancer, Lung OR Cancers, Lung OR Lung Cancers OR Pulmonary Cancer OR Cancer, Pulmonary OR Cancers, Pulmonary OR Pulmonary Cancers OR Cancer of the Lung OR Cancer of Lung)

#3 #1 or #2

#4 (ground glass opacity OR ground glass node OR ground glass nodule OR GGO OR GGN)

#5 #3 and #4
